# Serum Phthalate Levels and Time to Pregnancy in Couples from Greenland, Poland and Ukraine

**DOI:** 10.1371/journal.pone.0120070

**Published:** 2015-03-18

**Authors:** Ina Olmer Specht, Jens Peter Bonde, Gunnar Toft, Christian H. Lindh, Bo A. G. Jönsson, Kristian T. Jørgensen

**Affiliations:** 1 Department of Occupational and Environmental Medicine, Bispebjerg University Hospital, Copenhagen, Copenhagen NV, Denmark; 2 Department of Occupational Medicine, Aarhus University Hospital, Aarhus C, Denmark; 3 Division of Occupational and Environmental Medicine, Lund University, Lund, Sweden; University of Rennes-1, FRANCE

## Abstract

Phthalates are ubiquitous industrial chemicals that have been associated with altered reproductive function in rodents. Several human studies have reported an inverse association between male testosterone and phthalate levels. Our aim was to investigate time to pregnancy (TTP) according to serum levels of diethylhexyl phthalate (DEHP) and diisononyl phthalate (DiNP) metabolites in both partners. In 2002-2004 we enrolled 938 pregnant women and 401 male spouses from Greenland, Poland and Ukraine. Six oxidized metabolites of DEHP and DiNP were summarized for each of the two parent compounds to provide proxies of the internal exposure. We used Cox discrete-time models to estimate fecundability ratios (FR) and 95% confidence intervals (95% CIs) for men and women according to their proxy-DEHP or -DiNP serum levels adjusted for a fixed set of covariates.

The FR was slightly elevated among women with high levels of DEHP (FR=1.14, 95% CI 1.00;1.30) suggesting a shorter TTP in these women. The FR was unrelated to DiNP in women, whereas the results for men were inconsistent pointing in opposite directions. First-time pregnant women from Greenland with high serum DiNP levels had a longer TTP. This study spanning large contrast in environmental exposure does not indicate adverse effects of phthalates on couple fecundity. The shorter TTP in women with high levels of DEHP metabolites is unexplained and needs further investigation.

## Introduction

The probability of conceiving in a given menstrual cycle can be affected by several factors including age, body mass index (BMI), tobacco smoking, and alcoholic beverages [34; 35]. Some studies have shown that environmental and occupational contaminants also can affect the time to pregnancy (TTP) [9; 12; 39]. Chemicals that influence TTP may subsequently influence birth outcomes, and studies have shown that maternal exposure to environmental contaminants may increase the age of menarche, menstrual cycle length, follicle numbers and TTP in the adult daughters [10; 14; 28] and adversely affect semen quality in sons [44].

Phthalates are ubiquitous, industrial chemicals found among others in building materials, toys, medical devices, rain clothes and cosmetics [11; 25; 26; 38]. Diethylhexyl phthalate (DEHP) and diisononyl phthalate (DiNP) belong to the heavy molecular weighted phthalates (ester side-chain lengths with five or more carbons) and their metabolites have been associated with altered reproductive development and function in rodents [22]. DiNP exposure has been associated with increased nipple retention, reduced anogenital distance, reduced sperm motility and increased sperm count in male rats, and masculinization of female dams [2], while DEHP has been shown to affect the function of Leydig cells in rats [27; 29]. Mono-2-ethylhexyl phthalate (MEHP, the primary DEHP metabolite) has been shown to induce ovarian toxicity by suppressing follicular development with decreased viability of follicles and apoptosis of granulosa cells, increased progesterone level and decreased levels of androstenedione, testosterone, and estradiol [21].

Several epidemiologic studies have reported altered reproductive hormones in men exposed to DEHP or DiNP metabolites [23; 31; 32; 36; 40], but still the evidence both in animal and human studies are conflicting [1]. Occupational phthalate exposure was associated with a prolonged TTP (TTP>6 month) in a study based on a job-exposure matrix of chemical exposure in the work place [7]. However, a study investigating paternal indoor air exposure to occupational DEHP found no association with couple TTP [33]. Similar, a recent study investigating couple fecundity did not find affected TTP by DEHP metabolites, they did however observe a longer TTP for men exposed to metabolites of benzylbutyl phthalates and dimethyl phthalates[6].

We have previously reported a negative association between serum levels of DEHP and DiNP metabolites and male testosterone, semen volume and total sperm count [40]. In the current study we will use the same INUENDO cohort of pregnant women and their spouses from Greenland, Poland and Ukraine to investigate TTP according to serum levels of DEHP and DiNP metabolites in both partners.

## Materials and Methods

In accordance with the Declaration of Helsinki, all participants signed an informed consent and the study was approved by local ethical committees; Polish Bioethical Committee, Ethical Committee for Human Research in Greenland and the Commission on Ethics and Bioethics Kharkiv National Medical University in Ukraine.

### Study populations

This study is based on the INUENDO cohort. The study population has previously been described in detail [41]. In brief, between May 2002 and February 2004 we consecutively invited a total of 3,833 pregnant women and their male partners at the first routine antenatal care visit at local hospitals in 19 cities and settlements throughout Greenland, a large central hospital in Warsaw, Poland, and from three hospitals and eight antenatal clinics in Kharkiv, Ukraine. In total 1,710 (44.6%) pregnant women agreed to participate with the country-specific participation rates being 90% (598/665) in Greenland, 68% (472/690) in Poland, and 26% (640/2478) in Ukraine. Among the male spouses, approximately 200 men in each region were consecutively enlisted. Both partners were at least 18 years old and born in the country of study, they were interviewed and gave a blood sample [41].

The participants were face-to-face interviewed from a structured interview questionnaire. The questions used to establish the TTP of the women were:

*“Leading up to this pregnancy*, *when was it that you started having sexual intercourse without using any birth control to prevent pregnancy*?*”* Month:______ Year:______. *We now call this the “Starting time”*.

*“How long was it from that “Starting Time” until you became pregnant*? *(the date you became pregnant is the date you conceived) how long*?*”* Weeks:_______ and/or Months:_____ an/or Years:______ [41].

Additionally, the questionnaire also contained a question on the date when the couple stopped using contraception [41].

Because of unplanned pregnancies and pregnancies occurring while the couples were using contraception we had in-valid or missing information about TTP for 568 women. A further 204 women did not provide a blood sample. Thus, the final population of women providing both interview data and a blood sample was overall 938, with 448, 203 and 287 women from Greenland, Poland and Ukraine, respectively. For the male study population, we had information regarding their couple TTP for 411 men. However, we had no blood sample for 10 men, leaving a final male study population of 401 men with 160, 146 and 95 men from Greenland, Poland and Ukraine, respectively.

### Measurements of phthalates

Phthalate metabolites were analyzed in serum samples by liquid chromatography-tandem mass spectrometry system (LC-MS/MS; UFLCXR, Shimadzu Corporation, Kyoto, Japan) at the Department of Occupational and Environmental Medicine at Lund University, Sweden. Analyses included the three secondary oxidized metabolites of DEHP [2-ethyl-5-hydroxy-hexyl phthalate (5OH-MEHP), 2-ethyl-5-oxyhexyl phthalate (5oxo-MEHP) and 5-carboxy-mono-2-ethylpenty phthalate (5cx-MEPP)], and the three metabolites of DiNP [mono-4-methyl-7-hydroxy-octyl phthalate (7OH-MMeOP), mono-4-methyl-7-oxo-octyl phthalate (7oxo-MMeOP) and mono-4-methyl-7-carboxyheptyl phthalate (7cx-MMeOP)].

The analytical method is the same which previously has been described in details by Lindh *et al*. (2012) for Perfluorinated compounds. In brief, the serum was added with labeled internal standards for all analyzed metabolites and glucoronidase to remove glucoronic acid. The proteins were then precipitated by organic solvent, the samples centrifuged and the supernatant injected into the LC-MS/MS equipment. The quality of the analysis was checked by including chemical blank samples and in-house quality control in all analyzed sample batches. Moreover, each sample was analyzed three times in three different analytical batches. The imprecision in the analyzed control sample was 8% for 5OH-MEHP, 9% for 5oxo-MEHP, 18% for 5cx-MEPP, 8% for 7OH-MMeOP, 7% for 7oxo-MMeOP and 19% for 7cx-MMeOP. The limits of detection (LODs) were improved in the analyses of the women compared to the men (Table A in S1 File) mainly because the levels of contamination in the mobile phases were reduced. Values <LOD were imputed by the maximum likelihood single imputation method [30]. The laboratory is a European reference laboratory for urinary phthalate metabolite analyses for all the included compounds (www.eu-hbm.info/democophes).

### Statistical analysis

The total serum concentrations of the DEHP metabolites (5OH-MEHP, 5oxo-MEHP and 5cx-MEPP) as well as the DiNP metabolites (7OH-MMeOP, 7oxo-MMeOP and 7cx-MMeHP) were calculated by summing their molecular weights to provide an estimate of the internal concentration of their primary metabolites, MEHP and monoisononyl phthalate (MiNP), respectively. Thus, Proxy-MEHP refers to proxy estimates of the DEHP primary metabolite, MEHP, while Proxy-MiNP refers to proxy estimates of the DiNP primary metabolite, MiNP.

We analyzed the association between phthalate exposure (both single metabolites and summed metabolites) and TTP specifically for each country. We categorized measures of female or male serum concentrations of Proxy-MEHP and Proxy-MiNP into country-specific tertiles with the lowest category being the reference category. In addition, we studied the association between TTP and phthalates on a continuous natural logarithm transformed scale specifically for each country and for a pooled sample comprising the entire cohort of 938 women or the 401 men, since it was not appropriate to investigate a pooled sample divided into tertiles because of different phthalate levels in each country. We calculated fecundability ratios (FRs) with 95% confidence intervals (95% CIs) using a modified Cox regression model that handled the TTP in months, the underlying time scale, as a discrete scale. The FR represents the probability of conceiving during a time period (e.g., one month or one menstrual cycle) within one group compared to the probability in the reference group, or in the analysis of continuous natural logarithm transformed proxies, the FR represents the probability of conceiving if the phthalate levels increased 2.7 times, corresponding to one unit increase of the natural logarithm of the concentration due to the irrational number *e*. TTP was censored after 13 months of trying to conceive to take digit preference into account (proportionally more women recall TTPs of 12 months compared to 11 and 13 months) and to avoid increased risk of medical intervention bias [45]. In the analysis of female phthalate levels the FRs were adjusted for maternal age (<25, 25–29, ≥30 years), smoking status in pregnancy (yes, no), frequency of sexual intercourse (daily, ≥1 per week, <1 per week, unknown frequency), parity status (primiparous, 1, ≥2 children), maternal BMI (<20, 20–24, ≥25) as well as gestational week of interview and blood sampling (gestational week 1–20, 21–30, 31–42). In men the FRs were adjusted for paternal age (<25, 25–29, ≥30 years), paternal BMI (<20, 20–24, ≥25) and maternal age (<25, 25–29, ≥30 years). Additionally we adjusted the pooled FR analysis for country (Greenland, Poland, Ukraine). Possible confounding variables were selected based on *a priori* considerations of their role as known or potential risk factors. FRs less than 1 denote reduction in fecundity or longer TTP, and FRs greater than 1 denote a shorter TTP.

Infertility odds ratios (ORs) and 95% CIs were calculated using logistic regression with Firth correction [18]. Women with TTPs >13 months were categorized as infertile while women with TTPs ≤13 months were fertile. The OR analysis was adjusted for the same covariates as in the Cox regression model.

To test the robustness of the main findings we made sensitivity analyses where we restricted to a) primiparous women and b) men with primiparous spouses. The tertile categorization in these analyses was based on the original Proxy-MEHP and Proxy-MiNP tertile levels used in the main analysis. We also made FR and OR analyses of the single metabolites where >70% of the measurements were above the LOD [30]. Two of the six metabolites, 5oxo-MEHP and 7oxo-MMeOP, were for that reason excluded in the analysis of the men.

Two-sided statistical tests were applied and p-values <0.05 and 95% CI excluding unity were considered statistically significant. The statistical analysis was conducted in SAS Enterprise, version 5.1 for Windows (SAS Institute Inc., Cary, NC, USA).

## Results

The median TTP was 4 [interquartile range (IQR) 2–8], 4 (IQR 2–9) and 5 (IQR 3–13) months for women from Greenland, Poland and Ukraine, respectively. The proportion of infertile couples (TTP >13 months) was 14% in Greenland, 16% in Poland and 20% in Ukraine. Characteristics of the men and women in the study are shown in Table 1. The Ukrainian women were the youngest with a mean age of 23 years, followed by women from Greenland (25 years) and Poland (28 years). Women from Greenland were more likely to have a BMI >25, smoke and have one or more children. In Greenland, the women reported the birth control pill the most frequent contraceptive method used previous to the index pregnancy, whereas the majority in Poland used condom and in Ukraine withdrawal was by the majority used as the last contraceptive methods (Table 1). In all three countries the majority of couples had intercourse at least once a week.

**Table 1:** Demographic information for women and men in the three study populations.

	Greenland		Poland		Ukraine	
	women	men	women	men	women	men
N	448	160	203	146	287	95
Age, mean (years)	26	31	28	31	23	27
Body mass index (%)						
<20	12	3	34	2	33	5
20–25	51	43	56	42	54	56
>25	36	53	9	55	13	38
Smoking (%)	73	74	18	27	23	64
No children (%)	31	31	91	95	79	71
One child (%)	31	36	7	4	18	24
>One child (%)	38	31	1	0	2	3
Gestational week of blood sampling (%)						
1–20	32	31	1	0	39	41
21–30	34	33	15	16	21	13
31–42	28	29	76	75	32	39
Last contraceptive methods (%)						
Safe periods	1	1	13	12	34	31
Withdrawal	4	4	12	11	32	36
Coil	24	31	1	1	2	5
Birth control pill	63	58	33	34	6	3
Condom	8	6	38	39	26	25
Jelly cream or foam	0	0	3	3	1	0
Frequency of sexual intercourse (%)						
Daily	44	44	17	16	21	23
At least once a week	44	44	68	70	37	36
Less than once a week	7	6	10	11	6	8
Don’t know	5	7	4	3	37	33

Men from Greenland and Poland had a mean age of 31 years, while men from Ukraine had a mean age of 27 years. The majority of men from Greenland and Poland had BMI>25, whereas in Ukraine most men had BMI between 20 and 25. The proportions of male smokers were 27%, 64% and 74% in Poland, Ukraine and Greenland, respectively.

In Table 2 the serum levels of phthalates are shown for each country and for the pooled sample. The percentages below LOD are showed for each metabolite in each country (Table 2). Overall, the pooled study population had higher serum levels of Proxy-MEHP compared to Proxy-MiNP. Between countries, men and women from Ukraine had the highest serum levels of Proxy-MEHP, whereas those from Greenland had the highest Proxy-MiNP serum levels. For all single metabolites and in each country, men had higher serum levels than women.

**Table 2:** Serum levels of DEHP and DiNP proxies and single metabolites in men and women.

Women	Greenland (n = 448)				Poland (n = 203)				Ukraine (n = 287)				Overall (n = 938)
	%<LOD	Mean	Median	Range	%<LOD	Mean	Median	Range	%<LOD	Mean	Median	Range	Median
Proxy-MEHP (pM)	-	6.41	5.09	1.01–75.02	-	5.70	4.63	1.41–40.23	-	7.60	5.32	0.87–88.42	4.98
5OH-MEHP	0	0.94	0.72	7.53–0.13	0	0.51	0.41	4.64–0.08	0	0.78	0.44	9.71–0.03	0.52
5oxo-MEHP	0	0.15	0.12	1.72–0.03	0.5	0.12	0.11	0.98–0.02	1.7	0.15	0.10	3.53–0.02	0.11
5cx-MePP	0	0.83	0.95	13.41–0.12	0	1.11	0.91	7.75–0.18	0	1.37	0.90	20.51–0.19	0.75
Proxy-MiNP (pM)	-	3.17	1.73	0.31–53.84	-	1.75	1.28	0.37–33.14	-	2.97	0.75	0.13–97.20	1.40
7OH-MMeOP	0	0.32	0.25	1.76–0.03	1.5	0.16	0.11	1.32–0.00	10.1	0.11	0.04	9.57–0.00	0.13
7oxo-MMeOP	9.2	0.03	0.02	0.55–0.00	6.9	0.03	0.02	0.59–0.00	13.2	0.05	0.01	3.45–0.00	0.01
7cx-MMeHP	1.6	0.66	0.24	17.2–0.01	0.5	0.37	0.27	8.67–0.02	1.4	0.81	0.19	22.90–0.02	0.23

Men	Greenland (n = 160)				Poland (n = 146)				Ukraine (n = 95)				Overall (n = 401)
Proxy-MEHP (pM)	-	9.78	8.16	2.56–80.52	-	8.79	7.69	3.98–27.33	-	15.52	8.73	3.10–123.66	7.97
5OH-MEHP	0	1.20	1.01	5.05–0.25	0	0.69	0.58	2.73–0.21	3.2	1.01	0.53	7.49–0.12	7.98
5oxo-MEHP	44.4	0.23	0.21	0.91–0.04	59.6	0.18	0.16	0.60–0.04	55.8	0.27	0.18	2.44–0.04	0.69
5cx-MePP	0	1.52	1.11	20.68–0.29	0	1.79	1.51	7.40–0.58	0	3.43	1.81	33.13–0.53	0.19
Proxy-MiNP (pM)	-	3.80	2.86	0.73–31.35	-	3.21	2.63	1.04–23.47	-	8.30	2.04	0.66–151.93	2.63
7OH-MMeOP	1.3	0.36	0.31	2.25–0.05	4.8	0.27	0.20	1.47–0.04	19.0	0.43	0.16	6.81–0.04	2.63
7oxo-MMeOP	54.4	0.04	0.03	0.32–0.00	61.6	0.03	0.02	0.44–0.00	66.3	0.13	0.02	2.82–0.00	0.22
7cx-MMeHP	0	0.80	0.52	7.40–0.13	0	0.73	0.57	5.56–0.21	0	2.09	0.50	41.97–0.12	0.02

%<LOD = % below limits of detection.

### Fecundability and infertility ratios in women and men and levels of Proxy-MEHP and Proxy—MiNP

As reported in Table 3, the women’s FR point estimates for highest tertile exposure of Proxy-MEHP were above one in all three countries (Greenland FR = 1.32, Poland FR = 1.20, Ukraine FR = 1.26) and the FR was significantly increased for women from Greenland compared to the lowest tertile (FR = 1.32, 95% CI 1.01; 1.78), suggesting a shorter TTP for the highly exposed women. Proxy—MiNP in women was not associated with the FR in the country-specific analyses. Proxy-MEHP and-MiNP were not associated with OR of infertility in women.

**Table 3:** Adjusted^a^ fecundability ratios (FR) and odds ratio for infertility (OR) according to female Proxy-MEHP and—MiNP by country, and association between TTP and phthalates on a continuous logarithm transformed scale for each country and for pooled samples.

Women		Tertiles		Continues data	
Proxy-MEHP (median pM)	N	FR (95%CI)	OR (95%CI)	FR (95%CI)	OR (95%CI)
**Overall**	938			1.14(1.00,1.30) *	0.86(0.64;1.14)
**Greenland**	448			1.24(1.01;1.53) *	0.89(0.54;1.41)
3.01		1	1		
5.10		1.31 (0.99;1.73)	0.52 (0.27;1.02)		
9.07		1.32 (1.01;1.78)*	0.73 (0.39;1.36)		
**Poland**	203			1.13(0.78;1.64)	0.79(0.38;1.65)
3.24		1	1		
4.64		1.00 (0.64;1.55)	1.12 (0.50;2.75)		
7.48		1.20 (0.78;1.85)	0.61 (0.23;1.59)		
**Ukraine**	287			1.08(0.88;1.33)	0.95(0.62;1.47)
2.80		1	1		
5.32		1.14 (0.80;1.64)	0.96 (0.45;2.04)		
10.39		1.26 (0.87;1.82)	0.73 (0.34;1.58)		
**Proxy-MiNP tertiles (median pM)**					
**Overall**	938			0.98(0.90;1.06)	1.09(0.90;1.31)
**Greenland**	448			0.92(0.80;1.05)	1.33(0.98;1.80)
1.06		1	1		
1.74		1.28 (0.97;1.68)	0.57 (0.27;1.18)		
3.70		0.92 (0.70;1.22)	1.54 (0.82;2.89)		
**Poland**	203			0.16(0.86;1.58)	0.72(0.36;1.43)
0.80		1	1		
1.29		1.09 (0.71;1.70)	0.85 (0.34;2.11)		
2.40		1.20 (0.80;1.86)	0.61 (0.24;1.56)		
**Ukraine**	287			0.95(0.84;1.08)	1.09(0.84;1.42)
0.40		1	1		
0.75		1.12 (0.78;1.60)	1.02 (0.48;2.20)		
2.63		0.98 (0.69;1.40)	1.18 (0.56;2.48)		

^a^Adjusted for maternal age, smoking in pregnancy, frequency of sexual, parity status, maternal BMI, gestational week of interview, blood sampling and frequency of sexual intercourse.

* = p<0.05.

Similar results were observed, when the proxies were analyzed on a continuous log-transformed scale, significant FRs above one were observed in women from Greenland (FR = 1.24, 95% CI 1.01;1.53) and when the three study sites were pooled (FR = 1.14, 95% CI 1.00; 1.30) (Table 3).

In the analysis of male serum levels of proxies of MEHP and MiNP and FRs and ORs of infertility, associations were inconsistent between the three countries. Men from Greenland with high levels of Proxy-MEHP had a FR of 1.90 (95% CI 1.16; 3.09) compared to men with low levels (Table 4). In contrast, men from Ukraine with medium and high levels of Proxy-MEHP had FRs of 0.38 (95% CI 0.18; 0.77) and 0.71 (95% CI 0.36–1.37), respectively. Likewise for the ORs of infertility the point estimates for men with medium and high levels of Proxy-MEHP in Greenland were below one (ORs of 0.81 and 0.53, respectively) while ORs for men from Poland and Ukraine were above one (ORs between 2.62 and 1.10). For Proxy-MiNP men from Greenland with high serum levels had an increased FR of 1.73 (95% CI 1.05; 2.85). None of the ORs of infertility in relation to Proxy-MiNP reached levels of statistical significance and the point estimates for the three countries were inconsistent pointing in opposite directions (Table 4). Also, in the analysis of the men based on the continuous log-scale, similar results were observed, although with an overall significant positive association of Proxy-MiNP (FR = 1.23, 95% CI 1.04;1.45) (Table 4).

**Table 4:** Adjusted^a^ fecundability ratios (FR) and odds ratio for infertility (OR) according to male Proxy-MEHP and—MiNP by country, and association between TTP and phthalates on a continuous logarithm transformed scale for each country and for pooled samples.

Men		Tertiles		Continues data	
Proxy-MEHP (median pM)	n	FR (95%CI)	OR for infertility	FR(95%CI)	OR(95%CI)
**Overall**	401			1.17(0.94;1.45)	0.82(0.50;1.34)
**Greenland**	160			1.59(1.27;2.24)*	0.42(0.16;1.10)
4.77		1	1		
8.19		1.21 (0.75;1.96)	0.81 (0.27;2.43)		
13.50		1.90 (1.16;3.09)*	0.53 (0.17;1.65)		
**Poland**	146			0.87(0.53;1.43)	1.19(0.39;3.61)
5.54		1	1		
7.69		0.92 (0.55;1.51)	1.73 (0.55;5.45)		
11.27		0.70 (0.43;1.17)	2.31 (0.75;8.15)		
**Ukraine**	95			1.05(0.72;1.54)	0.83(0.36;1.88)
6.16		1	1		
8.90		0.38 (0.18;0.77)*	2.62 (0.51;13.42)		
18.73		0.71 (0.36;1.37)	1.10 (0.22;5.45)		
**Proxy-MiNP tertiles (median pM)**					
**Overall**	401			1.23(1.04;1.45)*	0.84(0.57;1.23)
**Greenland**	160			1.55(1.09;2.19)*	0.73(0.31;1.71)
1.73		1	1		
2.87		1.27 (0.78;2.06)	0.44 (0.13;1.45)		
4.64		1.73 (1.05;2.85)*	0.71 (0.23;2.25)		
**Poland**	146			1.09(0.71;1.68)	0.94(0.37;2.40)
1.82		1	1		
2.63		0.97 (0.58;1.60)	0.96 (0.31;2.95)		
4.28		0.96 (0.73;1.62)	1.21 (0.40;3.64)		
**Ukraine**	95			1.18(0.93;1.49)	0.96(0.57;1.64)
1.12		1	1		
2.06		0.84 (0.42;1.66)	2.67 (0.55;13.06)		
6.53		1.90 (0.95;3.79)	0.63 (0.15;2.78)		

^a^Adjusted for paternal age, paternal BMI and maternal age.

* = p<0.05.

Results for primiparous women were similar as for all women, with exception of a significant FR below null for women from Greenland exposed to Proxy-MiNP when analyzed on the continuous log-scale (FR = 0.72, 95% CI 0.54; 0.95). The FR of 0.72(0.54;0.95), indicates that if a first time pregnant woman from Greenland got her Proxy-MiNP serum levels increased 2.7 times, her probability of conceiving in a menstrual cycle decreases with 28%(5%;46%) (Table 5). Also, when primiparous women from Greenland were analyzed divided into tertiles, the association was significant (Table B in S1 File). Results for men with a primiparous partner were similar to results of the entire male study population (Table 5).

**Table 5:** Stratified analysis of first time pregnancies. Adjusted^a^,^b^ association between TTP and phthalates on a continuous logarithm transformed scale for each country and for pooled samples.

First time pregnant Women	n	FR(95% CI)	OR(95%CI)
**Proxy-MEHP**			
Overall	552	1.06(0.90;1.25)	0.99(0.68;1.45)
Greenland	138	1.33(0.88;2.01)	0.96(0.34;2.74)
Poland	187	1.14(0.79;1.66)	0.77(0.36;1.65)
Ukraine	227	0.05(0.84;1.32)	0.99(0.62;1.60)
**Proxy-MiNP**			
Overall	552	0.95(0.85;1.06)	1.13(0.88;1.45)
Greenland	138	0.72(0.54;0.95)*	2.64(1.15;6.06) *
Poland	187	1.31(0.96;1.79)	0.50(0.23;1.11)
Ukraine	227	0.93(0.81;1.08)	1.19(0.88;1.62)
**Men with first time pregnant wives**			
**Proxy-MEHP**			
Overall	255	1.23(0.92;1.63)	0.73(0.37;1.45)
Greenland	49	13.16(4.24;40.84) *	0.11(0.01;0.84) *
Poland	139	0.89(0.54;1.48)	0.99(0.32;3.11)
Ukraine	67	1.14(0.72;1.82)	0.66(0.21;2.04)
**Proxy-MiNP**			
Overall	255	**1.26(1.02;1.56)**	0.66(0.38;1.15)
Greenland	49	**2.10(1.02;4.32)**	0.65(0.16;2.68)
Poland	139	1.38(0.87;2.19)	0.57(0.20;1.69)
Ukraine	67	1.22(0.90;1.94)	0.72(0.32;1.65)

^a^Analysis of women were adjusted for maternal age, smoking in pregnancy, frequency of sexual, parity status, maternal BMI, gestational week of interview, blood sampling and frequency of sexual intercourse.

^b^Analysis of men were adjusted for paternal age, paternal BMI and maternal age.

* = p<0.05.

Results of single metabolites of all men and women and for primiparous women and men with primiparous wives were comparable with results of proxies.

Similar trends for associations showed in Tables 3–5 where observed in crude analysis (Table C in S1 File).

## Discussion

The time it takes to become pregnant from when a woman starts to have regular unprotected intercourse can be used as a measure of couple fecundity. The chance of conceiving in a cycle is heterogeneous, varying from a zero chance in sterile couples to a very high chance in highly fecund couples. In this large study of 938 pregnant women and 401 male spouses we showed that TTP was not consistently associated with phthalates among men or women at the three study sites. Women and men from Greenland with high serum levels of Proxy-MEHP had a FR above 1 compared to those with low levels. In contrast, we found that men from Ukraine exposed to medium levels of Proxy-MEHP had a FR below 1, corresponding to a longer couple TTP. When only first-time pregnant women were investigated, we observed a FR below 1 in women from Greenland with high serum levels of Proxy-MiNP and a higher risk for infertility.

The mainly null findings for men in our study support the results from a recent study investigating urinary levels of 14 phthalate metabolites and bisphenol A in 501 couples [6]. The authors did not find statistically significant associations between DEHP metabolites and TTP, but they did observe longer TTP among wives of men who had high levels of monomethyl phthalate, mono-n-butyl phthalate and monobenzyl phthalate [6]. An occupational study of male workers exposed to DEHP in ambient air (269 pregnancies with TTP information among 153 men) was likewise unable to show prolonged TTP in the workers’ wives [33]. The internal exposure in that study was unknown since DEHP was measured in air and not urine, serum or semen. A study based on a job exposure matrix found an increased risk of TTP > 6 month in women with probable phthalate exposure [7]. The estimated job exposure was not reflecting the intensity, duration or frequency of exposure, and the TTP was dichotomized in TTPs ≤ and > 6 months. Similarly, a Danish register based study observed an increased incidence of infertility treatment among women working in the plastic industry [19]. In this occupation the workers were exposed to a variety of synthetic macromolecules, molecular monomers as well as different organic and inorganic materials, possibly including the two plasticizers, DEHP and DiNP [19].

Our study has some limitations, especially the exposure time window in TTP studies and the use of TTP as a measure of couple fecundity. Because DEHP and DiNP have short biological half-lives in the range of hours to days and do not accumulate, it is unknown whether the levels measured during pregnancy reflect the levels in the period when the couples initiated their first pregnancy attempts. A study of 50 men and women observed considerable within-subject variability of urinary levels of phthalate metabolites during eight consecutive days [13]. The authors concluded that exposure assessments should not be based on single-individual urine measurements. Another study concluded, in spite of both substantial day-to-day and month-to-month variability in each individual's urinary phthalate metabolite levels, that a single urine sample was predictive of each subject's exposure over 3 months [17]. The physiological changes during pregnancy might influence absorption, distribution, metabolism or the excretion of phthalates. A study investigated the variability of urinary phthalate metabolites before and during pregnancy in 113 women who provided overall 853 urine samples. They found within-women variation of concentrations of DEHP metabolites, but the absolute differences before and after pregnancy were relatively small [5]. In our study population phthalates were measured in serum and not urine. It is unknown if the within-subject variation of phthalate metabolites is the same as in urine. We have previously shown that the serum phthalate levels did not vary with time of blood sampling in men [40], which is in contrast to what has been observed in studies using urine samples [31]. Since the within-subject variation from conception to the blood sampling is unknown in our cohort, we do not know how often persons might move from one exposure group to another. A high degree of within-subject shifting between exposure groups (e.g., from tertile 1 to 2) over shorter periods of time would blur the true associations with TTP. Population studies worldwide have consistently demonstrated phthalate exposure in more than 95% of the participants [46], which indicate chronic exposure to the phthalates, so it is unlikely that our study populations were unexposed during the period before conception.

There are however some disadvantages with the use of serum instead of urine that recently have had much attention [8]. If the serum sample becomes contaminated with the ubiquitous di-esters of phthalate the lipase activity of serum will split the di-esters into mono-esters. The concentrations of mono-esters in serum are therefore not reliable measures of exposure, contrary to urine that has no lipase activity [16]. Therefore, we did not investigate the mono-esters MEHP and MiNP or any other phthalate mono-ester. We analyzed the oxidized phthalate metabolites for which there is no possible contamination from the parent phthalates. Another disadvantage with the use of serum is that the metabolite levels in serum are much lower than in urine. However, with our method most samples were above the LODs. On the other hand, the serum levels might be a better estimate of the internal dose than the urinary levels.

To investigate if our measures of phthalate levels in serum were valid we correlated the concentrations of phthalates in serum with concentrations in urine both from a group of young Swedish men. Except for a poor correlation of 5oxo-MEHP in serum and urine (r_S_ = 0.32) the levels of the other single phthalate metabolites were highly correlated in serum and urine, indicating that serum concentrations of metabolites can be used as biomarkers for human exposure [40]. Urine is rarely stored in bio-banks, therefore precise methods to investigate phthalate metabolites in serum is important.

Biased reporting of TTP is not likely to be a major problem in this study since the women were enrolled during pregnancy and reporting was blinded towards phthalate metabolite concentrations in blood. The majority of couples had intercourse daily or at least once a week, which considerably optimizes their chance of becoming pregnant. In our study 34, 46 and 294 women in Greenland, Poland and Ukraine, respectively, got pregnant while using contraception or by accident, and did not provide a TTP. These women may be more fecund than others, and therefore subfecund women may be overrepresented among those providing a valid TTP [3]. The high proportion of contraceptive failures in Ukraine is probably not due to higher couple fecundity in these couples, but rather related to their choice of contraceptive methods (withdrawal and safe periods). We investigated if phthalate levels differed in the women and men with valid TTP and without, the levels did not differ (results not shown). Further, we do not believe that our results are hampered by bias related to accidental pregnancies since they were excluded from the analysis. A further limitation of our study is that we only enrolled couples who achieved pregnancy. Thus, it was not possible to investigate whether DEHP or DiNP are associated with couple sterility. Cross-sectional studies of DEHP and DiNP in infertility clients do not report higher exposure levels of phthalates [20; 42]. Also, occupationally exposed men, who are highly exposed, do not show sterility or more impaired fertility, so we do not believe that DEHP and DiNP is associated with sterility [33; 36; 37].

A potential selection bias could be that we enrolled not only first time pregnancies, but also multiparous couples. As a sensitivity analysis we investigated primigravida women. In first time pregnant women from Greenland we observed that longer TTP was associated with higher serum levels of Proxy-MiNP. Studies have shown that primigravida women have longer TTP than women with more children [24; 34]. We can therefore not exclude that couples with past-pregnancy experience were better to plan the present pregnancy since they already had some knowledge about their fertility status.

The low participation rate in Ukraine was a consequence of the recruitment procedure where contact between potential participants and the project team were managed by approximately 30 medical doctors at the hospitals and antenatal clinics. With this large organization a high level of information and encouragement to participate was not possible. Demographic and reproductive information were obtained from a sample of 605 of those Ukrainian women that declined participation in the study. Only average age was slightly lower among non-participating women [22.8 (SD 2.4) versus 24.9 (SD 2.8) years], while the average number of children in the two groups were similar [4]. If the low participation rate in Ukraine is reflecting preferential participation of more or less fecund couples, bias is expected to be minimal since the phthalate levels were unknown for the invited population

DEHP is anti-androgenic in animal models, and we have in a previous publication shown a negative association between DEHP metabolites and testosterone in men [40]. Whether phthalates can affect human female fecundity is unknown, but *in vitro* studies have shown that DEHP and MEHP can inhibit antral follicular growth and estradiol production [15; 21]. A resent case-control study of women with and without polycystic ovary syndrome (PCOS) reported higher levels of some phthalates, including MEHP, in PCOS patients. The anti-androgenic effect of the phthalates might therefore be relevant in women [43].

Reasons for the observed FR above 1 in men and women from Greenland who were highly exposed to Proxy-MEHP could be lifestyle. Since DEHP is found in plastics, women with high DEHP serum levels might have a different lifestyle than women with lower levels. Such lifestyle differences might be essential for the differences in TTP between couples with low and high DEHP levels.

## Conclusion

This study does not indicate adverse effects of phthalates on couple fecundity. The reason for the observed shorter TTP in women with high levels of DEHP metabolites is unknown, but the suggested association between DEHP exposure and lifestyles associated to TTP and the anti-androgenic effect of the phthalates should be investigated in future studies.

## Supporting Information

S1 File This file contains Tables A, B, and C. Table A, Limits of detection (LODs) for men and women. Table B, First-time pregnant women. Fecundability ratio (FR) and odds risk ratio for infertility (OR) in first-time pregnant women and their levels of proxy-DEHP and—DiNP by country. Table C, Crude associations. Crude associations between TTP and phthalates on a continuous logarithm transformed scale for each country and for pooled samples.
(DOCX)
