# Supplementary material for: Serum Phthalate Levels and Time to Pregnancy in Couples from Greenland, Poland and Ukraine
Source: PLoS One. 2015 Mar 18;10(3):e0120070. doi: 10.1371/journal.pone.0120070 (PMC4364890; doi:10.1371/journal.pone.0120070)
Supplement: S1 File — Table A, Limits of detection (LODs) for men and women. Table B, First-time pregnant women. Fecundability ratio (FR) and odds risk ratio for infertility (OR) in first-time pregnant women and their levels of proxy-DEHP and—DiNP by country. Table C, Crude associations. Crude associations between TTP and phthalates on a continuous logarithm transformed scale for each country and for pooled samples. (DOCX) [file pone.0120070.s001.docx]

| **Table A:** **Limits of detection (LODs) for men and women.** | | | | |
| --- | --- | --- | --- | --- |
|  |  |  | LOD men (ng/ml) | LOD women (ng/ml) |
| 5OH-MEHP | |  | 0.2 | 0.01 |
| 5oxo-MEHP | |  | 0.2 | 0.02 |
| 5cx-MEPP | |  | 0.1 | 0.007 |
| 7OH-MMeOP | |  | 0.1 | 0.01 |
| 7oxo-MMeOP | |  | 003 | 0.005 |
| 7cx-MMeHP | |  | 0.1 | 0.03 |

# S1 File: Supporting Tables

| **Table B: First-time pregnant women.** Fecundability ratio (FR) and odds risk ratio for infertility (OR) in first-time pregnant women and their levels of proxy-DEHP and –DiNP by country. | | | | |
| --- | --- | --- | --- | --- |
|  |  | **Women** | |  |
| **Proxy-MEHP tertiles (nM)** | **n** | **FR** | **95%CI** | **OR for infertilitet** |
| **Greenland** | 138 |  |  |  |
| 0.001-0.004 |  | 1 |  | 1 |
| 0.004-0.006 |  | 1.09 | (0.65;1.84) | 0.79(0.20;3.14) |
| 0.006-0.075 |  | 1.16 | (0.70;1.91) | 1.29(0.35;4.72) |
| **Poland** | 187 |  |  |  |
| 0.001-0.004 |  | 1 |  | 1 |
| 0.004-0.006 |  | 1.10 | (0.71;1.73) | 0.95(0.37;2.41) |
| 0.006-0.040 |  | 1.33 | (0.78;1.94) | 0.60(0.22;1.62) |
| **Ukraine** | 227 |  |  |  |
| 0.0009-0.004 |  | 1 |  | 1 |
| 0.004-0.007 |  | 1.12 | (0.75;1.66) | 1.02(0.43;2.39) |
| 0.007-0.088 |  | 1.25 | (0.83;1.88) | 0.74(0.30;1.82) |
| **Proxy-MiNP tertiles (nM)** | | | | |
| **Greenland** | 138 |  |  |  |
| 0.0003-0.001 |  | 1 |  | 1 |
| 0.001-0.002 |  | 0.86 | (0.52;1.44) | 4.77 (0.64;35.86) |
| 0.002-0.054 |  | **0.52** | **(0.30;0.90)*** | **16.18 (2.12;123.23)*** |
| **Poland** | 187 |  |  |  |
| 0.0004-0.001 |  | 1 |  | 1 |
| 0.001-0.002 |  | 1.14 | (0.73;1.81) | 0.79 (0.32;1.99) |
| 0.002-0.033 |  | 1.44 | (0.91;2.26) | 0.45 (0.16;1.23) |
| **Ukraine** | 227 |  |  |  |
| 0.0001-0.0006 |  | 1 |  | 1 |
| 0.0006-0.001 |  | 1.19 | (0.80;1.76) | 0.87 (0.36;2.12) |
| 0.001-0.097 |  | 0.91 | (0.61;1.36) | 1.25 (0.53;2.97) |
| CI = 95% confidence interval, *=p<0.05 | | | | |

| **Table C: Crude associations.**  Crude associations between TTP and phthalates on a continuous logarithm transformed scale for each country and for pooled samples. | | |
| --- | --- | --- |
| **All Women** | **n** | **Crude FR(95%CI)** |
| **Proxy-MEHP** |  |  |
| Overall | 938 | **1.13(1.00;1.27)*** |
| Greenland | 448 | 1.19(0.98;1.45) |
| Poland | 203 | 1.03(0.72;1.43) |
| Ukraine | 287 | 1.14(0.93;1.39) |
| **Proxy-MiNP** |  |  |
| Overall | 938 | 1.00(0.93;1.08) |
| Greenland | 448 | 0.88(0.77;1.01) |
| Poland | 203 | 1.07(0.81;1.42) |
| Ukraine | 287 | 1.01(0.90;1.13) |
| **All Men** |  |  |
| **Proxy-MEHP** |  |  |
| Overall | 401 | 1.06(0.86;1.29) |
| Greenland | 160 | **1.52(1.09;2.13)*** |
| Poland | 146 | 0.81(0.51;1.30) |
| Ukraine | 95 | 0.97(0.69;1.38) |
| **Proxy-MiNP** |  |  |
| Overall | 401 | 1.13(0.97;1.33) |
| Greenland | 160 | **1.52(1.09;2.10)*** |
| Poland | 146 | 0.99(0.67;1.47) |
| Ukraine | 95 | 1.05(0.84;1.31) |
